# Inhibition of HtrA2 alleviated dextran sulfate sodium (DSS)-induced colitis by preventing necroptosis of intestinal epithelial cells

**DOI:** 10.1038/s41419-019-1580-7

**Published:** 2019-04-24

**Authors:** Chong Zhang, Andong He, Shuai Liu, Qiaoling He, Yiqin Luo, Zhilan He, Yujiao Chen, Ailin Tao, Jie Yan

**Affiliations:** 1 The Second Affiliated Hospital, The State Key Laboratory of Respiratory Disease, Guangdong Provincial Key Laboratory of Allergy & Clinical Immunology, Guangzhou Medical University, 510260 Guangzhou, China; 2 The First Affiliated Hospital, Guangdong Pharmaceutical University, 510006 Guangzhou, China

**Keywords:** Necroptosis, Ulcerative colitis

## Abstract

Necroptosis of intestinal epithelial cells has been indicated to play an important role in the pathogenesis of inflammatory bowel disease (IBD). The identification of dysregulated proteins that can regulate necroptosis in dextran sulfate sodium (DSS)-induced colitis is the key to the rational design of therapeutic strategies for colitis. Through tandem mass tag (TMT)-based quantitative proteomics, HtrA2 was found to be downregulated in the colon of DSS-treated mice. UCF-101, a specific serine protease inhibitor of HtrA2, significantly alleviated DSS-induced colitis as indicated by prevention of body weight loss and decreased mortality. UCF-101 decreased DSS-induced colonic inflammation, prevented intestinal barrier function loss and inhibited necroptosis of intestinal epithelial cells. In vitro, UCF-101 or silencing of HtrA2 decreased necroptosis of HT-29 and L929 cells. UCF-101 decreased phosphorylation of RIPK1 and subsequent phosphorylation of RIPK3 and MLKL during necroptosis. Upon necroptotic stimulation, HtrA2 translocated from mitochondria to cytosol. HtrA2 directly interacted with RIPK1 and promoted its degradation during a specific time phase of necroptosis. Our findings highlight the importance of HtrA2 in regulating colitis by modulation of necroptosis and suggest HtrA2 as an attractive target for anti-colitis treatment.

## Introduction

Inflammatory bowel diseases (IBD), namely Crohn’s disease and ulcerative colitis, affect about 3.7 million people in the USA and Europe^1^. However, its etiology remains elusive, as complex interactions between genetic susceptibility, microbial dysbiosis, and environmental factors are involved in its pathogenesis^2^. Intestinal epithelium provides a physical barrier that modulates microbial colonization and prevents their penetration of the epithelium^3^. Epithelial cell death is a hallmark of intestinal inflammation and leads to intestinal barrier disruption, which contributes to the pathogenesis of IBD^4^. Necroptosis, a newly recognized programmed cell death, of intestinal epithelial cells led to disruption of the intestinal barrier and resulted in spontaneous colitis or terminal ileitis in mice^5,6^. necrosulphonamide (NSA), an mixed lineage kinase domain like pseudokinase (MLKL) inhibitor, inhibited necroptosis of intestinal epithelial cells^7^. Moreover, Increased necroptosis was also found in colon tissues of CD and UC patients^8^. Although hard evidence for a contribution of necroptosis in human IBD still remains limited, intervening in necroptosis has been indicated as a promising therapeutic strategy for IBD. However, the role of deregulated genes that contribute to necroptosis in IBD remains largely unexplored.

Necroptosis is typically considered a highly pro-inflammatory mode of cell death, due to release of intracellular “damage-associated molecular patterns” that promote inflammation^9^. Necroptosis is a caspase-independent cell death and can be initiated by death receptors including TNFR1, TLR3, and TLR4^9,10^. Signal transduction during necroptosis has been well studied in the context of TNF-α. Upon TNF-α stimulation, RIPK1, FADD, and CYLD are recruited to TNFR1 to form a protein complex. Subsequent deubiquitylation and phosphorylation events lead to RIPK1 phosphorylation and activation^11,12^. When caspase-8 activity is absent or inhibited, the phosphorylated RIPK1 regulates the formation of a necrosome that consists of RIPK1, RIPK3, and MLKL^13^. Via RIP homotypic interaction motif-domain (RHIM) interactions, RIPK1 promotes oligomerization and subsequently phosphorylation of RIPK3^14,15^. MLKL is then recruited to the RIPK1/RIPK3 complex and phosphorylated by p-RIPK3. Phosphorylated MLKL forms oligomers and translocates to the intracellular plasma membrane where it binds to phosphatidylinositol lipids and cardiolipin, leading to the formation of pores and finally disrupting cellular membrane integrity^16^. As an essential factor for necroptosis in the context of TNF-α, RIPK1 is reported to regulate necroptosis positively in a kinase-dependent manner, and negatively in a kinase-independent manner whereby the scaffolding function of the RHIM domain prevents ZBP1 from activating RIPK3 and thus represses necroptosis^14,17,18^.

HtrA2 is a serine protease located in mitochondria and involved in apoptosis regulation^19^. Upon apoptotic stimuli, HtrA2 translocates from mitochondria to cytosol, where it binds to and cleaves IAPs, thus releasing caspases from their natural inhibitors^20^. Independently, HtrA2 can cleave anti-apoptotic proteins ped/pea 15 and Hax-1 and thereby promote apoptosis^21,22^. In addition to apoptosis, HtrA2 is also reported to have a role in necroptosis. In IL-13 deprivation induced cell death, HtrA2 was able to cleave RIPK1 and enhanced cell death in a caspase-independent manner^23^. According to the references, HtrA2 promotes necroptosis in a serine protease-dependent manner^24,25^. UCF-101, a serine protease inhibitor of HtrA2, could inhibit TNF-α plus Z-VAD induced necroptosis in neutrophils, L929Ts, and Jurkat I42 cells, as well as TNF-α plus Z-VAD and cycloheximide induced necroptosis in HT-29 cells^24,25^. Nonetheless, the exact mechanism for HtrA2 in necroptosis regulation and IBD pathogenesis still remains unknown and needs further investigation.

Based on high throughput proteomic analysis, we found significant downregulation of HtrA2 in colons of dextran sulfate sodium (DSS)-treated mice. Moreover, the pathological symptoms and animal motility were ameliorated by UCF-101 treatment. Mechanism investigation suggested that HtrA2 could interact with RIPK1, promote its degradation and finally enhance necroptosis. Our study raises the possibility of HtrA2 as a potential therapeutic target for anti-colitis treatment.

## Results

### HtrA2 is downregulated in colons of DSS-treated mice

**Fig. 1:**
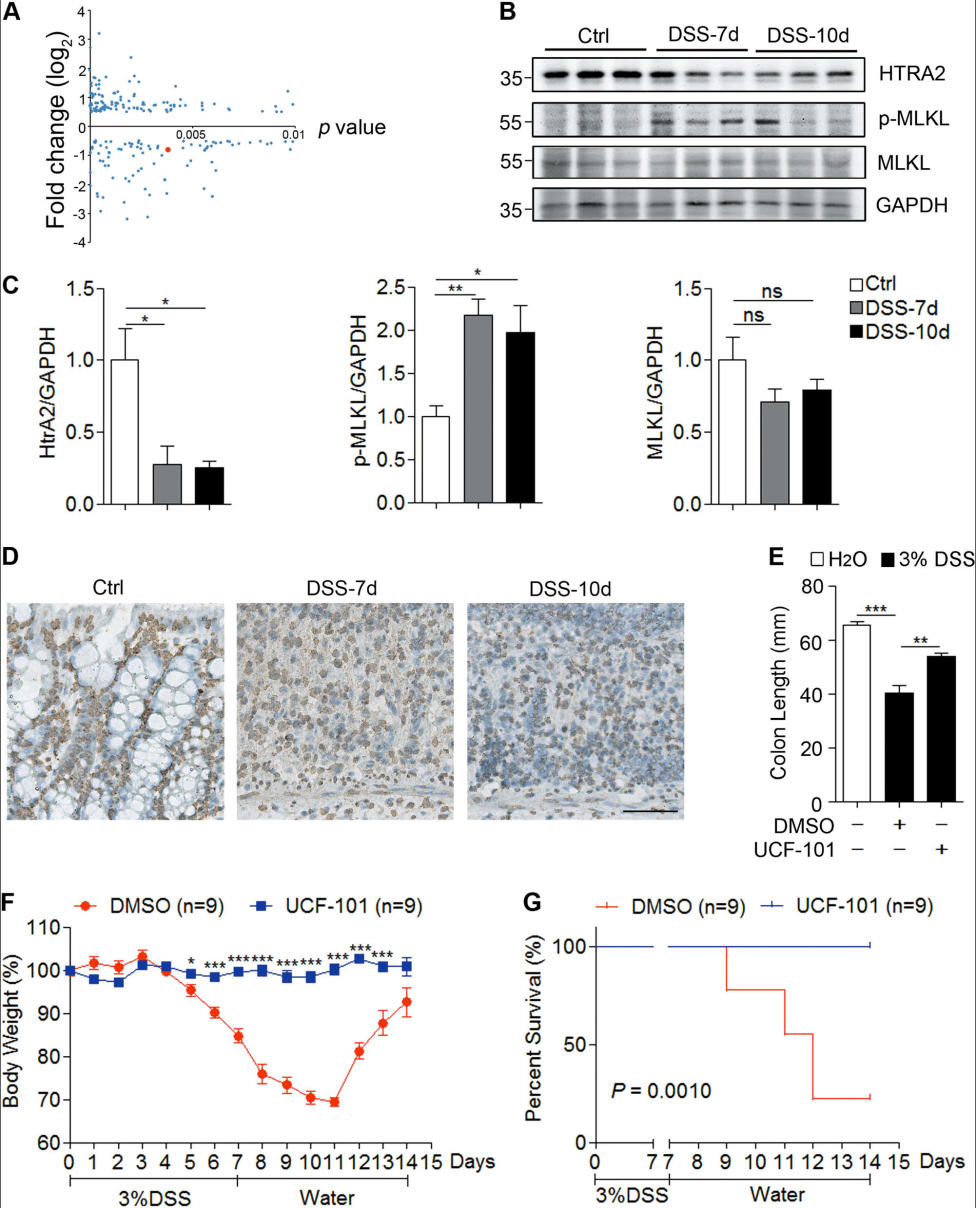
Pharmacological inhibition of HtrA2 ameliorated DSS-induced colitis. **a** Differentially expressed proteins in colons of control and DSS-treated mice. Three percent DSS was administered in drinking water to C57BL/6 mice for 7 days and replaced with fresh water thereafter. On day 10, colons were collected and protein levels were measured by quantitative proteomics. Each dot represents one protein. HtrA2 is indicated by red dot. *X* axis represents *P* value and *Y* axis represents fold change of colonic protein level between control and DSS-treated mice. *n* = 3 mice/group. **b**–**d** HtrA2 expression was decreased in colon of DSS-treated mice. Three percent DSS was used to induce colitis as described in **a**. On day 7 and day 10, colons were collected to analyze the protein levels of HtrA2, MLKL, phosphorylated MLKL, and GAPDH by immunoblotting with corresponding antibodies (**b**) or immunohistochemical (IHC) staining with anti-HtrA2 antibody (**d**). Densitometric analysis of immunoblotted proteins (**c**). Scale bar, 50 μm. **e**–**g** Three percent DSS was used to induce colitis as described in **a**, and UCF-101 (10-μm mol/kg mice) or DMSO was injected intraperitoneally every day for 10 days. Mice were killed on day 10 to measure the colon length (**e**); and body weight (**f**), and survival rate (**g**) was determined. In **c**, **e**, **f**, data are presented as means ± SEM. *P *<* 0.05; **P *<* 0.01; ***P *<* 0.001 (two-tailed unpaired Student’s *t* test)

To identify important proteins that are dysregulated in the inflamed colon that could be used as novel potential therapeutic targets, we utilized a DSS-induced colitis mouse model for quantitative proteomics. Control mice were given distilled water for 10 days while DSS-treated mice were given 3% DSS for 7 days that was replaced with distilled water for the following 3 days. Based on daily monitoring, the DSS-induced mice slowly began to lose weight and have bloody stools. On day 7, the symptoms were most severe and some of the mice died. The surviving mice recovered gradually and eventually returned to normal. Thus, we harvested the colon tissues on day 7 and day 10 to detect protein levels by tandem mass tag (TMT) quantitative proteomics. Differentially expressed proteins were used for the Kyoto encyclopedia of genes and genomes analysis (Supplementary Figs. 1 and 2). Several pathways were significantly enriched, including oxidative phosphorylation, complement, and coagulation cascades. Compared with the distilled water-treated control mice, HtrA2 protein levels were significantly decreased in the colons of DSS-treated mice (Supplementary Fig. 1 and Fig. 1a). In addition, the downregulation of HtrA2 in the colons of DSS-treated mice was confirmed by immunoblotting and immunohistochemical (IHC) staining (Fig. 1b–d). These data show that the protein level of HtrA2 was significantly reduced as colitis progressed. Further study is needed to determine whether the downregulation of HtrA2 is the cause of colitis development or whether the negative feedback protective mechanism is initiated by the colon tissue.

### Pharmacological inhibition of HtrA2 ameliorates DSS-induced colitis

To determine the role of HtrA2 in DSS-induced colitis, UCF-101 was used to inhibit the serine protease activity of HtrA2 in vivo. In the DSS-induced colitis mouse model, UCF-101 was given daily by intraperitoneal injection from day 0 to day 9. Compared to the control treatment (DMSO) in which DSS treatment led to a rapid body weight loss from day 5 to day 12, UCF-101 completely blocked body weight loss (Fig. 1f). Furthermore, UCF-101 dramatically reduced DSS-induced mortality and shortening of colon length (Fig. 1g, e). Taken together, these results imply that inhibition of HtrA2 prevents DSS-induced colitis in mice, suggesting that downregulation of HtrA2 is a protective mechanism utilized by the host in the context of colitis.

### UCF-101 decreased inflammation in colon

**Fig. 2:**
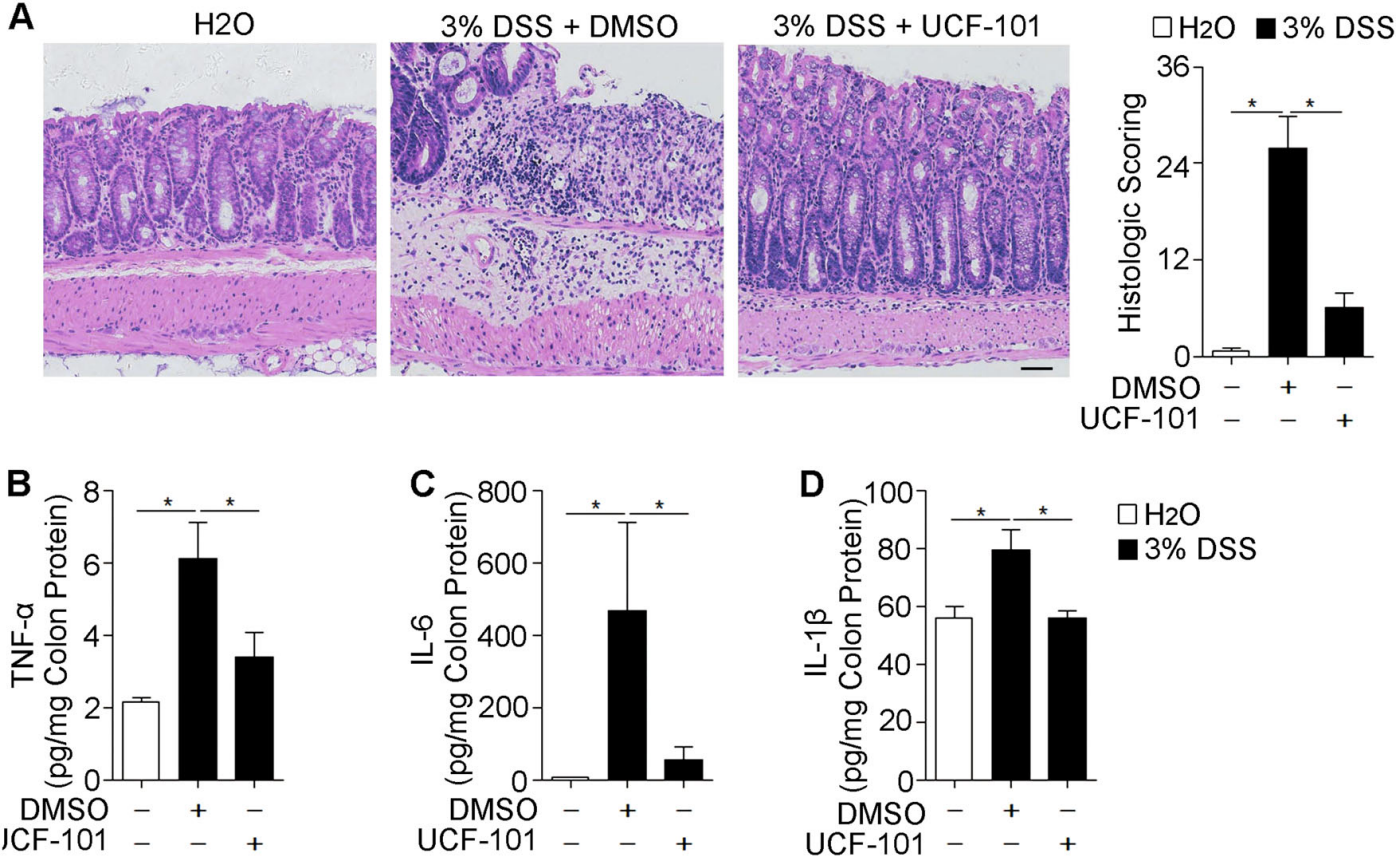
UCF-101 decreased inflammation in colons of DSS-treated mice. Three percent DSS was administered in drinking water to C57BL/6 mice for 7 days and replaced with fresh water thereafter. UCF-101 (10-μm mol/kg mice) or DMSO was injected intraperitoneally every day for 8 days. **a** Colon tissues from mice on day 8 were evaluated by H&E staining and histologic score analysis. Scale bar, 50 μm. **b**–**d** Total colon tissues on day 8 were extracted and 300 μg protein were used to measure TNF-α, IL-6, and IL-1β levels by ELISA. In (**a**–**d**), data are presented as means ± SEM. *P *<* 0.05 (two-tailed unpaired Student’s *t* test)

**Fig. 3:**
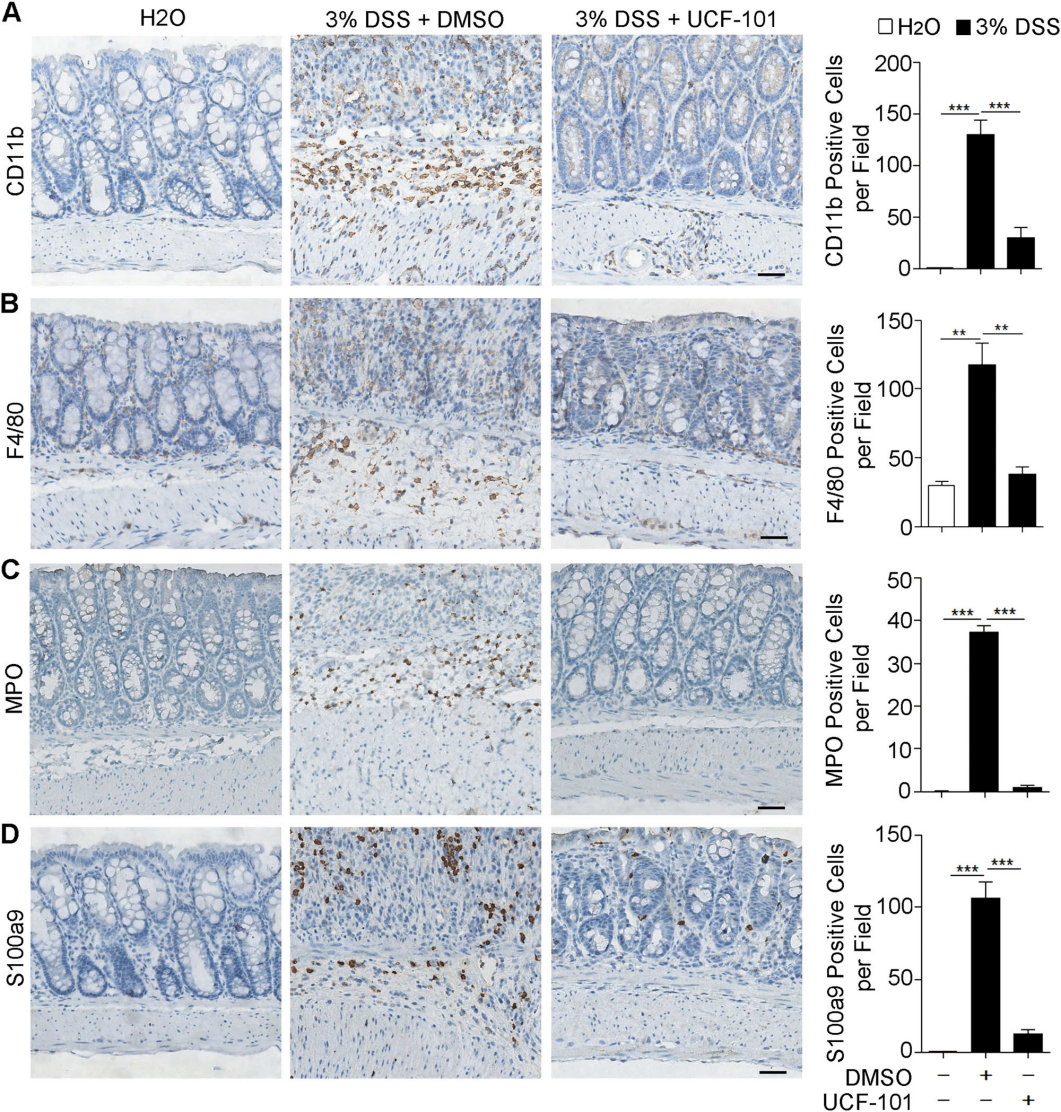
UCF-101 decreased the infiltration of CD11b, F4/80, MPO, and S100a9 positive cells in colons of DSS-treated mice. **a**–**d** Three persent DSS was administered in drinking water to C57BL/6 mice for 7 days and replaced with fresh water thereafter. UCF-101 (10μm mol/kg mice) or DMSO was injected intraperitoneally every day for 8 days. Colons were harvested and sections of colon tissues were immunohistochemically stained for CD11b (**a**), F4/80 (**b**), MPO (**c**) and S100a9 (**d**) with corresponding antibodies. Scale bar, 50 μm. Ten random fields (200×) were photographed for each section. The average number of positive cells per field is presented. In **a**–**d**, data are presented as means ± SEM. **P < 0.01; ***P < 0.001 (two-tailed unpaired Student’s *t* test)

Next, we examined whether UCF-101 decreased inflammation in DSS-induced colitis. HE staining showed that UCF-101 significantly decreased tissue damage and infiltration of inflammatory cells in colons of DSS-treated mice (Fig. 2a). Enhanced tissue damage in DMSO treated control mice was accompanied with augmented expression of pro-inflammatory cytokines, including TNF-α, IL-6, and IL-1β, which was significantly restrained in UCF-101 treated mice (Fig. 2b–d). In DSS-induced colitis, large numbers of myeloid cells (Cd11b positive), including macrophages (F4/80 positive) and neutrophils (MPO positive), infiltrated into the mucosa and epithelial layer of the damaged colon (Fig. 3a–c). The infiltration of Cd11b, F4/80, and S100a9 positive cells in the colon was dramatically suppressed in UCF-101-treated mice (Fig. 3a–c). The same phenomenon was observed with the infiltration of S100a9 positive cells, a marker of inflammation (Fig. 3d). Taken together, these results imply that the protease function of HtrA2 may play an important role in DSS-induced colonic inflammation.

### UCF-101 decreases intestinal barrier disruption and necroptosis in colons of DSS-treated mice

**Fig. 4:**
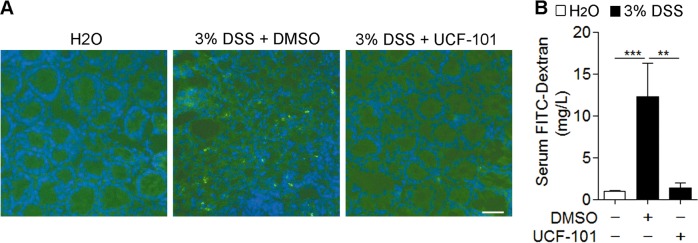
UCF-101 decreased intestinal barrier disruption in colons of DSS-treated mice. Three percent DSS was administered in drinking water to C57BL/6 mice for 7 days and replaced with fresh water thereafter. UCF-101 (10-μm mol/kg mice) or DMSO was injected intraperitoneally every day for 8 days. **a**, **b** Intestinal barrier permeability was detected by intragastrical injection of FITC-Dextran. **a** Colon tissues were sliced and representative images of colons from indicated groups were detected by fluorescent microscope. Scale bar, 50 μm. **b** FITC-Dextran levels in hemolysis-free serum from indicated groups were detected with spectrophotometer. In **b**, data are presented as means ± SEM. **P *<* 0.01; ***P *<* 0.001 (two-tailed unpaired Student’s *t* test). In **b**, data are presented as means ± SEM. **P *<* 0.01; ***P *<* 0.001 (two-tailed unpaired Student’s *t* test)

The intestinal barrier function is crucial for intestinal homeostasis. Intestinal inflammation is possibly due to the destruction of barrier function. We further explored the protective effect of UCF-101 on intestinal barrier function in colitis. Intestinal permeability was detected by intragastrical injection of FITC-Dextran tracker on day 8 of DSS induction. Increased FITC-Dextran was found in the colon and serum of control mice, but it was significantly reduced in UCF-101-treated mice (Fig. 4a, b), suggesting that the increased intestinal permeability seen after DSS induction could be diminished by UCF-101. Moreover, compared with the control treatment, UCF-101 significantly decreased the incidence of bacterial spreading to the spleens of DSS-treated mice (DMSO group: 3/6 versus UCF-101 group: 0/6, *p* = 0.0455; Supplementary Table 1). These data prove that treatment with UCF-101 can protect the barrier function of the colon in DSS-induced colitis.

**Fig. 5:**
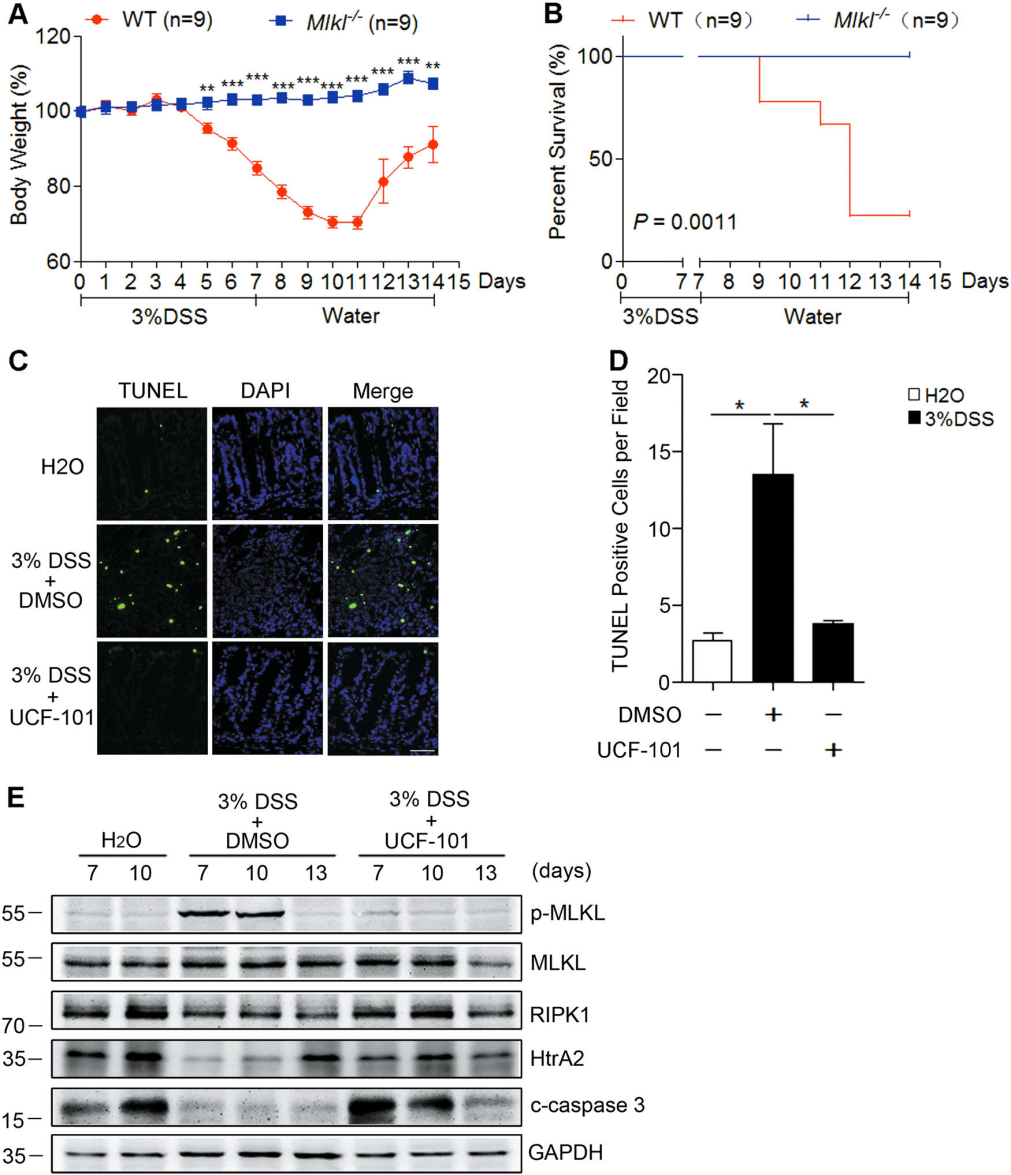
UCF-101 decreased necroptosis in colons of DSS-treated mice. **a**, **b** Three percent DSS was administered in drinking water to *Mlkl* deficient mice and their WT littermates for 7 days, and replaced with fresh water for the following days. Body weight (**a**) and mortality rate (**b**) were determined. **c**–**e** Three percent DSS was administered in drinking water to C57BL/6 mice for 7 days and replaced with fresh water thereafter. UCF-101 (10 μm mol/kg mice) or DMSO was injected intraperitoneally every day for 8 days. **c**, **d** Sections of colon on day 8 were subjected to TUNEL staining. Representative images (**c**) and numbers of TUNEL positive cells (**d**) are presented. **e** Colonic proteins on day 7, 10, and 13 were tested by immunoblotting to detect p-MLKL, MLKL, RIPK1 and cleaved caspase-3 with corresponding antibodies. GAPDH was used as an internal control. In **d**, data are presented as means ± SEM. *P *<* 0.05 (two-tailed unpaired Student’s *t* test)

Previous studies suggest that necroptosis of epithelial cells is an important process that leads to disruption of the intestinal barrier and contributes to the development of IBD. Based on our results, we found that compared with their littermate wild type mice, *Mlkl*^−/−^ mice were completely protected from DSS-induced colitis as indicated by prevention of body weight loss and reduced mortality (Fig. 5a, b), thus suggesting a critical role of necroptosis in DSS-induced colitis. Massive death of intestinal cells was found in DSS-treated wild type mice as shown by TUNEL staining, but treatment with UCF-101 significantly decreased TUNEL positive cells in the colon (Fig. 5c, d). Since TUNEL staining is not able to distinguish necroptosis from apoptosis, we further checked the evidence of necroptosis in the colon via immunoblotting with specific necroptotic and apoptotic markers; p-MLKL (promoter of necroptosis) and cleaved caspase-3 (indicator of apoptosis). In the colons of DSS-treated mice, p-MLKL was increased on day 7 and day 10, but little cleaved caspase-3 was detected (Figs. 1b and 5e), suggesting that necroptosis, but not apoptosis, contributed to DSS-induced colitis. As mice were observed to finally recover from DSS-induced colitis, immunoblotting results showed that p-MLKL decreased to basal levels on day 13 (Fig. 5e). In UCF-101 treated mice, much less p-MLKL was detected, demonstrating a suppression of necroptosis by UCF-101 (Fig. 5e). Interestingly, the level of cleaved caspase-3 was much greater in UCF-101-treated mice than in control mice (Fig. 5e). Collectively, all of these results suggest that UCF-101 can decrease the symptoms of colitis by preventing necroptosis of colonic epithelial cells and protecting intestinal barrier function.

### Inhibition of HtrA2 decreases necroptosis of epithelial cells in vitro

**Fig. 6:**
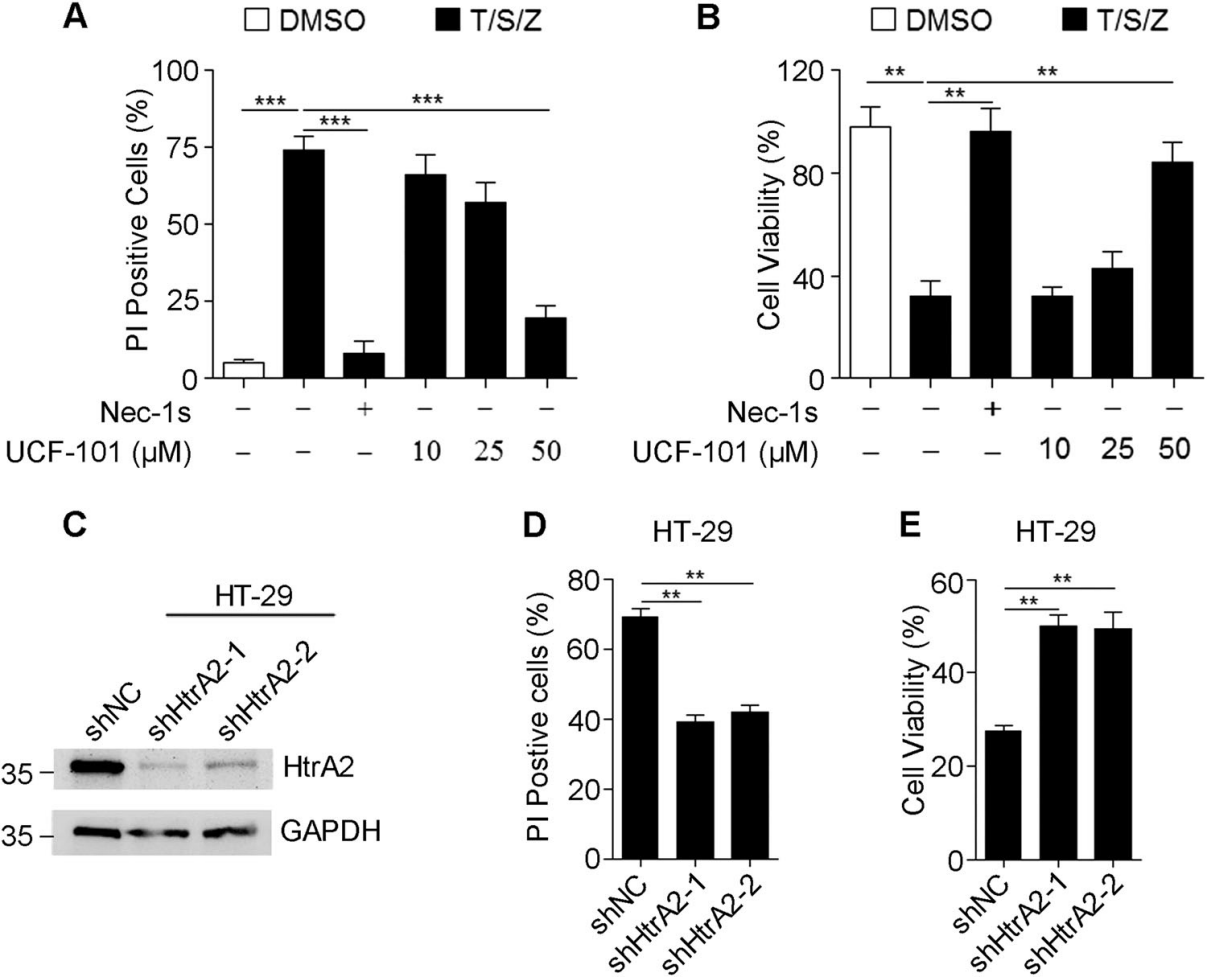
Inhibition of HtrA2 decreased necroptosis in TNF-α/Smac mimetic/Z-VAD (T/S/Z)-treated HT-29 cells. **a**, **b** HT-29 cells were pretreated with Nec-1s (10 μM) or different doses of UCF-101 for 1 h, followed by stimulation with TNF-α (20 ng/mL)/Smac mimetic (2 μM)/Z-VAD (25 μM) for 8 h. PI positive cells were analyzed by PI/Hoechst staining (**a**), and cell viability was determined by CCK8 analysis (**b**). **c**–**e** HT-29 cells were stably infected with lentiviruses carrying scramble shRNA (shNC) or two different shRNAs targeting two individual sites of HtrA2 (shHtrA2-1 or shHtrA2-2). HtrA2 protein levels were detected by immunoblotting (**c**). Indicated cells were stimulated with T/S/Z for 8 h. PI positive cells were analyzed by PI/Hoechst staining (**d**), and cell viability was determined by CCK8 (**e**). In **a**, **b**, **d**, **e**, data are presented as means ± SEM. **P *<* 0.01; ***P *<* 0.001 (two-tailed unpaired Student’s *t* test)

To find the mechanism by which HtrA2 regulates necroptosis, we treated HT-29, a type of colorectal adenocarcinoma cell, with TNF-α plus Smac mimetic and Z-VAD (T/S/Z) in vitro. T/S/Z-induced necroptosis of HT-29 cells was measured by PI/Hoechst staining and cell viability analysis. The necroptotic cell death was further confirmed using Nec-1s (RIPK1 inhibitor) as a positive control where Nec-1s treatment completely blocked T/S/Z-induced necroptosis of HT-29 cells (Fig. 6a, b and Supplementary Fig. 3). The results showed that necrosis induced with T/S/Z in HT-29 cells was decreased after treatment with UCF-101 in a dose-dependent manner (Fig. 6a, b and Supplementary Fig. 3). Similar results were obtained in L929 cells treated with T/S or T/Z (Supplementary Fig. 4). To confirm the role of HtrA2 in necroptosis, shRNAs against HtrA2 were used to decrease the protein level of HtrA2 (Fig. 6c). Consistent with the above observation, silencing of HtrA2 inhibited necroptosis of HT-29 cells as indicated by decreased PI positive cells and increased cell viability (Fig. 6d, e). These results suggest that HtrA2 contributes to necroptosis by its serine protease activity.

### HtrA2 contributes to necroptosis by degrading RIPK1

**Fig. 7:**
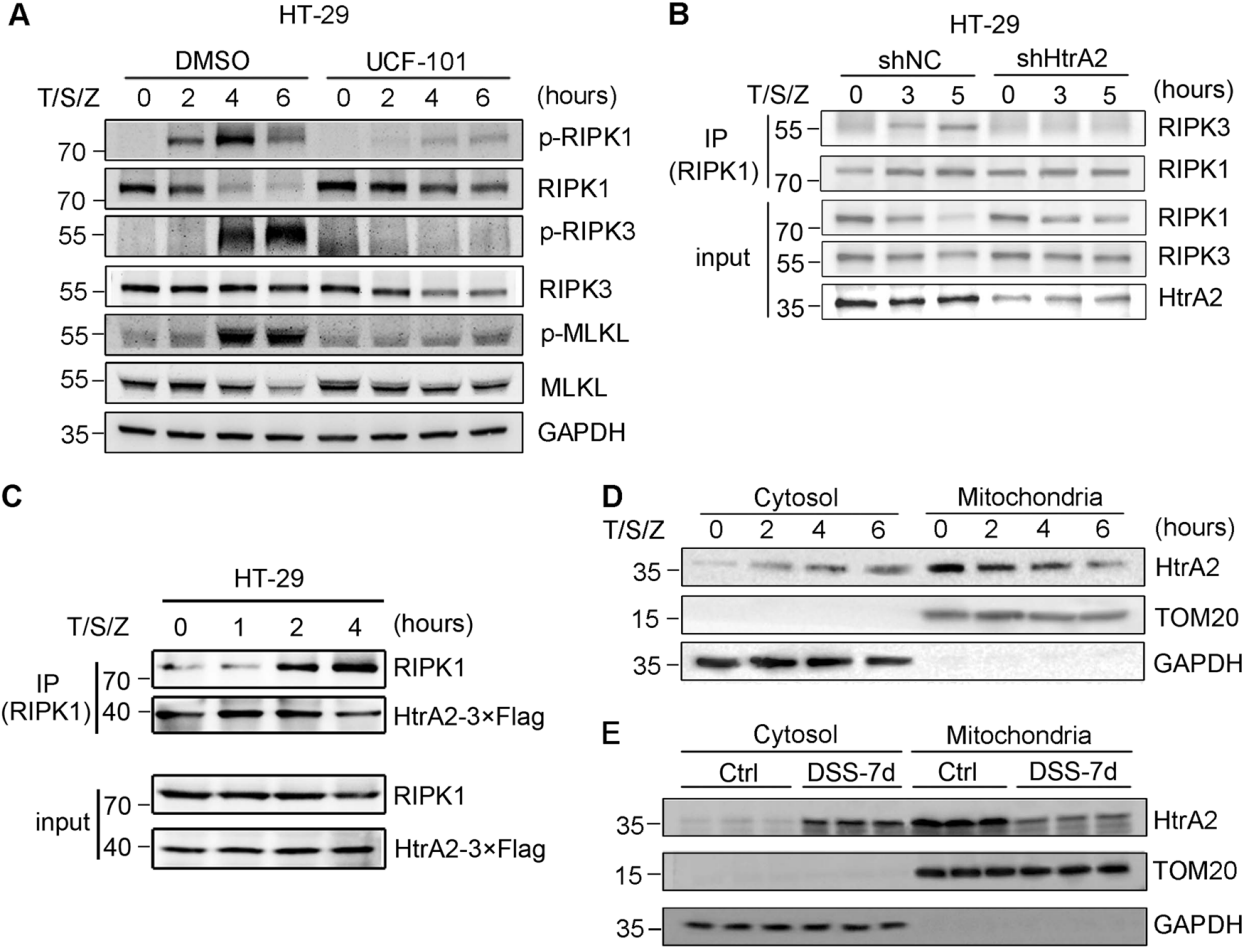
HtrA2 enhanced necroptosis by degrading RIPK1. **a** HT-29 cells were pretreated with UCF-101 (50 μM) for 1 h, followed by stimulation with T/S/Z for different times as indicated. Phosphorylation of RIPK1, RIPK3, and MLKL, as well as their protein levels, were analyzed by immunoblotting with corresponding antibodies. **b** Silencing of HtrA2 inhibited formation of necrosome in T/S/Z treated HT-29 cells. HT-29-shNC or HT-29-shHtrA2 cells were stimulated with T/S/Z for indicated times. The association between RIPK1 and RIPK3 was analyzed by immunoprecipitation with anti-RIPK1 antibody, followed by immunoblotting. **c** HT-29 cells stably expressing HtrA2-3× Flag fusion protein were stimulated with T/S/Z for indicated times. The association between RIPK1 and HtrA2-3× Flag was analyzed by immunoprecipitation with anti-Flag antibody, followed by immunoblotting. **d** HtrA2 translocated from mitochondria to cytosol during necroptosis. HT-29 cells were stimulated with T/S/Z for different times as indicated. Thereafter, the cytosol and the mitochondrial fractions were subjected to SDS-PAGE. Immunoblotting was performed with specific antibodies for the indicated proteins. **e** HtrA2 translocated from mitochondria to cytosol from mitochondria to cytosol during DSS-induced colitis. The cytosol and the mitochondrial fractions of colon tissues from normal mice (day 0) and DSS-treated mice (day 7) were subjected to SDS-PAGE. Immunoblotting was performed with specific antibodies for the indicated proteins. In d, e, antibodies to TOM20 and GAPDH served as controls for purity of mitochondrial and cytosolic fractions, respectively

To find the target of HtrA2, we examined the effect of UCF-101 on important factors involved in necroptosis, including RIPK1, RIPK3, MLKL, and their phosphorylated status. Immunoblotting results showed that HT-29 cells treated with T/S/Z showed an increase in p-RIPK1, p-RIPK3 and p-MLKL in a time-dependent manner (Fig. 7a). These necroptotic indicators were decreased when HT-29 cells were treated with UCF-101, a result consistent with the analysis of PI staining and cell viability (Fig. 6a, b and Supplementary Fig. 3). UCF-101 also decreased MLKL trimer formation in T/S-treated L929 cells, which is seen in the execution phase of necroptosis (Supplementary Fig. 5). Furthermore, silencing of HtrA2 decreased necrosome formation as indicated by decreased RIPK1/RIPK3 interaction in T/S/Z treated HT-29 cells (Fig. 7b). Moreover, immunoblotting results also showed that the total RIPK1 protein level was decreased upon T/S/Z stimulation, but degradation of RIPK1 was inhibited by UCF-101 treatment (Fig. 7a, the second panel). The same phenomenon was detected in colons of DSS-treated mice (Fig. 5e, the third panel). These data suggested that RIPK1 might be the target of HtrA2 during necroptosis.

To confirm that RIPK1 is the direct target of HtrA2, we detected their interaction by Co-IP. A HT-29 cell line which expressed HtrA2 fused with 3× Flag was constructed by lentivirus (Supplementary Fig. 6). HtrA2 was immunoprecipitated with Flag antibody, and the coimmunoprecipitated proteins were detected by immunostaining with RIPK1 antibody. After T/S/Z stimulation, increased interaction between HtrA2 and RIPK1 was detected (Fig. 7c). These results suggest that HtrA2 enhances necroptosis by directly interacting with RIPK1 and promoting its degradation.

HtrA2 is located in mitochondria while RIPK1 is cytosolic protein. So we speculated that HtrA2 translocated from mitochondria to cytosol to interact with RIPK1. Upon T/S/Z stimulation, mitochondrial HtrA2 decreased while cytosolic HtrA2 increased (Fig. 7d). Consistently, HtrA2 translocated from mitochondria to cytosol during DSS treatment in vivo (Fig. 7e). Taken together, these results indicate that HtrA2 translocated from mitochondria to cytosol and promoted necroptosis by degrading RIPK1 during necroptotic stimulation.

## Discussion

In this article, we reveal that HtrA2 is an important regulator of necroptosis; it enhanced necroptosis by degrading RIPK1 in a serine protease dependent manner at a specific time phase. Inhibiting the protease function of HtrA2 by treatment with UCF-101 ameliorated DSS-induced colitis in vivo. Our findings indicate HtrA2 downregulation as a protective mechanism to suppress necroptosis of colonic epithelial cells and to maintain colon barrier function in DSS-induced colitis. Targeting HtrA2 may be a potential therapy for IBD treatment.

Necroptosis has been thought to play an important role in the pathogenesis of IBD^26^. However, previous evidence has not definitively demonstrated a critical role for necroptosis in DSS-induced colitis. According to published data, the RIPK1-RIPK3-MLKL signaling pathway is the critical regulatory mechanism for necroptosis^27^. Nec-1, a RIPK1 inhibitor, suppresses DSS-induced colitis, but it is also an inhibitor of indoleamine 2,3-dioxygenase, which contributes to development of colitis^28,29^. RIPK3 deficiency had no effect on or even exacerbated DSS-induced colitis since RIPK3 deficiency compromised injury-induced tissue repair by impairing the IL-1β, IL-23, and IL-22 cytokine cascade^30–32^. Herein, we provide direct evidence for the critical role of necroptosis in DSS-induced colitis. MLKL is a promoter of necroptosis and p-MLKL has been used for the detection of necroptosis^16,33^. In this study, we found that p-MLKL was significantly increased in colons of mice with DSS-induced colitis and that MLKL deficiency completely protected mice against DSS-induced colitis. All of these results suggest that necroptosis has a critical role in the pathogenesis of colitis.

A substantial number of studies have found that HtrA2 promotes apoptosis by degrading IAP and other anti-apoptotic proteins^19,20^. However, its role in necroptosis and IBD remains unclear. Herein, we found that HtrA2 was significantly downregulated in the colons of DSS-treated mice and was associated with the pathological process. Moreover, UCF-101 treatment in vivo depressed necroptosis rather than apoptosis, thus protecting intestinal barrier function and inhibiting inflammation in DSS-induced colitis. In vitro, HtrA2 deficiency or UCF-101 treatment inhibited T/S/Z-induced necroptosis in HT-29 cells and T/Z-induced necroptosis in L929 cells, respectively. Therefore, HtrA2 promotes necroptosis in a serine protease dependent manner and leads to epithelial damage of the colon in DSS-induced colitis.

Previous studies have shown that HtrA2 promotes apoptosis in vitro. However, we found UCF-101 did not inhibit apoptosis in DSS-induced colitis. We speculate that HtrA2 may not be involved in colitis-associated apoptosis. Moreover, UCF-101 significantly inhibited necroptosis in DSS-induced colitis. Our data suggested that activation of HtrA2 contributed to DSS-induced colitis by promoting necroptosis but not apoptosis.

Apoptosis is a major mechanism for shedding of intestinal epithelium and is important in maintaining tissue homeostasis. We found that apoptosis was decreased in colon of DSS-treated mice, which was reversed by treatment with UCF-101. Given the fact that apoptosis of senescent intestinal epithelial cells occurs at the top of the colonic crypts, the decreased apoptosis in DSS-treated mice may be explained by the lack of colonic crypts, which is severely damaged and lost during colitis development (Fig. 2a). When the damage of colonic crypts and colitis was prevented by UCF-101 treatment, apoptosis of intestinal epithelial cells returned to the normal level.

In this study, we are the first to report that HtrA2 promotes necroptosis by degrading RIPK1. As a key player in the induction of necroptosis, RIPK1 utilizes two opposing mechanisms in necroptosis regulation. It promotes necroptosis in a kinase dependent manner to further promote RIPK3 activation and MLKL phosphorylation^14^. On the other hand, RIPK1 negatively inhibits necroptosis in a kinase independent manner^14,18^. RIPK1 deficiency or kinase inactive mutation blocks necroptosis in the context of TNF-a induction^34^, suggesting a critical role of RIPK1 kinase activity in mediating necroptosis. RIPK1 could also inhibit necroptosis by preventing ZBP1 from activating RIPK3 through its RHIM domain^18^. Herein, our findings showed that RIPK1 was phosphorylated upon T/S/Z stimulation in a time-dependent manner. Interestingly, RIPK1 was gradually degraded during necroptosis, followed by phosphorylation of RIPK3. These findings suggest that RIPK1 is required for signaling transduction upon T/S/Z induction and degradation of RIPK1 further enhances necroptosis. Moreover, UCF-101 inhibited degradation of RIPK1 and subsequently decreased phosphorylation of RIPK3 and MLKL (Fig. 7a). Direct interaction between HtrA2 and RIPK1 during necroptosis was also demonstrated by Co-IP (Fig. 7c). These results imply that HtrA2 enhances necroptosis by degrading RIPK1 at a specific time phase. Furthermore, we found that HtrA2 translocated from mitochondria to cytosol during necroptosis in vitro and in vivo, suggesting that HtrA2 interacted with RIPK1 in cytosol. By contrast, two groups reported that HtrA2 does not exit from mitochondria during necroptosis. In their model, TNF-α + Z-VAD and TNF-α were used to induce necroptosis of human neutrophils and L929 cells^25,35^, respectively. This discrepancy may be caused by the different cells and stimulations used by our experiments. However, how HtrA2 is activated upon T/S/Z stimulation and the subsequent regulation of RIPK1 phosphorylation and degradation need further study.

Unexpectedly, as a promoter of necroptosis, HtrA2 was not increased in DSS-treated colon. Since TNF-α is increased in colitis and endogenous HtrA2 is able to mediate necroptosis, necroptosis can still happen even when HtrA2 is not increased during DSS treatment. Moreover, HtrA2 translocated from mitochondria to cytosol during DSS treatment, thus the cytosolic HtrA2 may be an active regulator of necroptosis. Since HtrA2 is downregulated in colitis and returned to normal level in UCF-101 treated mice, we propose that downregulation of HtrA2 is a protective mechanism to suppress necroptosis of colonic epithelial cells and to maintain colon barrier function in DSS-induced colitis.

In addition to the anti-necroptosis function of UCF-101 in vitro, we also found that UCF-101 could ameliorate DSS-induced colitis by preventing necroptosis of intestinal epithelial cells. Intestinal barrier breakdown, increased infiltration of inflammatory cells and production of pro-inflammatory cytokines are major characteristics of IBD^4,36^, but all of these symptom could be alleviated in DSS-induced colitis when mice were treated with UCF-101. This is the first time that treatment with UCF-101 has been shown to suppress intestinal epithelial permeability, infiltration of macrophages and neutrophils, and production of TNF-α, IL-6, and IL-1β in the colons of DSS-treated mice. In addition, UCF-101 decreased DSS-induced body weight loss, colon length shortening and mortality of mice. Although UCF-101 inhibited necroptosis and ameliorated colitis in vivo, which is consistent with the pro-necroptosis activity of HtrA2 in vitro, we can not preclude the nonspecific effect of UCF-101 in vivo. At present, the off-target effect of UCF-101 has been concerned^37^. According to the report, UCF-101 could promote the activation of transcription factors CHOP, ATF3, and some stress-activated signaling pathways such as SAPK/JNK and ERK, which were independent of HtrA2 inhibition. It is indicated that the pro-survival function of UCF-101 in vivo was not only dependent on HtrA2 inhibition, but also mediated with various stress-induced signaling pathways. Alternatively, it’s possible that cytoplasmic HtrA2 may have a pro-inflammatory effect that is inhibited by UCF-101, and UCF-101 may also inhibit the translocation of HtrA2 from mitochondria to cytosol. Therefore, the off-target effect of UCF-101 in vivo needs further exploration in the future. For the specific role of HtrA2 in necroptotic regulation and the relativity in DSS-induced colitis, the epithelial-specific HtrA2-knockout mice are needed for further investigation.

Taken together, we discover HtrA2 as a positive regulator of necroptosis and colitis by degrading RIPK1 and our results suggest UCF-101 as a potential candidate for anti-colitis therapy in the future.

## Materials and methods

### Cell lines

Human colorectal adenocarcinoma cell line HT-29 was maintained in McCoy’s 5a Medium Modified (GIBCO, USA) supplemented with 10% fetal bovine serum (FBS, GIBCO, USA), penicillin (100 U/mL) and streptomycin (100 U/mL). Mouse fibroblast L929 was maintained in Dulbecco’s modified Eagle’s medium (DMEM, GIBCO) supplemented with 10% FBS, penicillin (100 U/mL) and streptomycin (100 U/mL).

### Mice

*Mlkl*^−/−^ mice were a generous gift from Dr. Jiahuai Han (State key laboratory of Cellular Stress Biology and School of life sciences, Xiamen University, China). Heterozygous *Mlkl* mice were further bred for age-matched wild type littermate and *Mlkl* deficient homozygous experimental mice. C57BL/6J mice were purchased from Jinan Peng Yue Laboratory Animal Breeding Company Limited (China). All mice were housed in specific pathogen-free (SPF) facility with a 12:12-h light/dark cycle and ambient temperature of 22 ± 2 °C. All protocols involving animals were conducted in accordance with the Guide for the Care and Use of Laboratory Animals (NIH publications no. 80-23, revised 1996) and under the approval of the Ethical Committee of Guangdong Provincial Animal Experiment Center.

### Reagents

The antibodies used for immunoblotting included: mouse monoclonal antibody against GAPDH (RM2002, Beijing Ray, Beijing, China); rabbit monoclonal antibodies against HtrA2 (ab75982, Abcam, Cambridge, MA, USA), p-RIPK1 (65746, Danvers, MA, CST, USA), RIPK1 (3493, CST), p-RIPK3 (93654, CST), human p-MLKL (91689, CST), human MLKL (ab184718, Abcam), and mouse p-MLKL (ab196436, Abcam); rabbit polyclonal antibodies against RIPK3 (ab56164, Abcam) and mouse MLKL (ab172868, Abcam); and goat anti-mouse (R3001, Beijing Ray) or goat anti-rabbit (R3002, Beijing Ray) HRP-conjugated secondary antibody.

The antibodies used for IHC staining included: CD11b (ab133357, Abcam), S100a9 (73425, CST, USA), HtrA2 (ab75982, Abcam), MPO (ab9535, Abcam), and F4/80 (ab111101, Abcam).

Other reagents included: DSS (36,000–50,000 kD, MP Biomedicals, Santa Ana, CA, USA), UCF-101 (Cayman Chemical, USA), Nec-1s, BV-6, Z-VAD (Selleck, Houston, TX, USA), mouse TNF-α (R&D, Minneapolis, MN, USA), Cell Counting Kit-8 (CCK-8, MedChemExpress, Monmouth Junction, NJ, USA), FITC-dextran (4 kDa, Sigma, St. Louis, MO, USA).

### Induction of experimental DSS-induced colitis

Male C57BL/6 mice weighing 21–24 g were used. DSS (3% wt/vol) was administered in drinking water ad libitum for 7 days (from day 0 to day 7). DSS solution was replaced twice on day 2 and day 4. For UCF-101 intervention experiments, mice were injected intraperitoneally with UCF-101 (10-μm mol/kg mice, dissolved in distilled water containing 10% DMSO) or same amount of 10% DMSO as control, from day 0 to day 9^38^. Mice weight and survival were recorded daily.

For proteomic analysis, colon tissues from control mice and 3% DSS-treated mice (*n* = 3 for each group) were collected and colonic proteins were extracted using the cold acetone method. Proteins were then tryptic digested with sequence-grade modified trypsin at 37 °C overnight. The resultant peptide mixture was labeled with TMT tags. The combined labeled samples were subjected to a strong cation exchanger fractionation column connected with a high-performance liquid chromatography system. Peptide fractions were resuspended with 30 μl solvent C (water with 0.1% formic acid), separated by nanoLC and analyzed by on-line electrospray tandem mass spectrometry. The fusion mass spectrometer was operated in the data-dependent mode to switch automatically between MS and MS/MS acquisition. The mass spectrometry data were transformed into MGF files (The Materials and Geometry Format file type) with Proteome Discovery 1.2 (Thermo, Pittsburgh, PA, USA) and analyzed using Mascot search engine (Matrix Science, London, UK; version 2.3.2). The Mascot search results were averaged using medians and quantified. Proteins with a fold change >1.3 or <0.77 and with *a P* value <0.05 were considered statistically significant.

For histologic scoring, H&E stained colonic tissue sections were used^39^. Histologic scoring was performed based on the degree of epithelial damage and inflammatory infiltration into the mucosa, submucosa and muscularis/serosa (score 0–3). Each of the four scores was multiplied by 1–3 depending on whether the change was focal, patchy, or diffuse, respectively. A total scoring range of 0–36 per mouse was obtained by adding up the four individual scores.

### Measurement of intestinal permeability

The mice treated with DSS for 7 days were deprived of food for 4 h, given FITC-dextran (4 kDa, 0.6 mg/g body weight, dissolved in 0.1 ml PBS) intragastrically and hemolysis-free sera were collected 3 h later. Intestinal permeability correlates with fluorescence intensity of serum (excitation, 488 nm; emission, 520 nm; Multi-Mode Microplate Reader).

To detect the bacterial load in spleen, spleen lysates (100 mg/ml in PBS) were centrifuged for 3 min at 300 *g*. The same volume of each supernatant was plated on nonselective agar plates in five serial ten-fold dilutions. Colonies of bacteria were observed 24 h later. Results were calculated from at least eight plates prepared from each sample.

### IHC staining

As described previously^40^, mice subjected to different treatments were killed and the same part of their colons were fixed in 4% paraformaldehyde for 12 h. The tissues were sliced to 5 µm thickness and deparaffinized with xylene, rehydrated through graded ethanol, followed by quenching of endogenous peroxidase activity in 0.3% hydrogen peroxide, and antigen retrieval by microwave heating in 10 mM citrate buffer (pH 6.0) for HtrA2, CD11b, and S100a9 or in EDTA buffer (pH 9.0) for MPO and F4/80. Sections were incubated at 4 °C overnight with rabbit polyclonal antibody against CD11b, S100a9, HtrA2, MPO, and F4/80, then immunostained by the ChemMate DAKO EnVision Detection Kit, Peroxidase/DAB, Rabbit/Mouse (DakoCytomation, Denmark). Subsequently, sections were counterstained with hematoxylin and mounted in non-aqueous mounting medium.

To detect the number of CD11b, F4/80, MPO, or S100a9 positive cells, ten random fields (200×) of each section were photographed to calculate the positive cells. The average numbers of positive cells per field are presented.

### Immunoblotting

Twenty miocrograms cell protein or 50 µg tissue protein were separated in a 10% polyacrylamide gel and transferred to a methanol activated PVDF membrane (Millipore, MA, USA). The membrane was blocked for 1 h in Tris-buffered saline plus Tween-20 (TBST) containing 3% bovine serum albumin, and then immunoblotted subsequently with primary and secondary antibodies. The protein level was detected using a Pierce ECL western blotting substrate (Thermo, USA). The molecular weight (kD) of each protein was indicated on the left side of the band.

### Measurement of cytokine secretion

For detecting cytokine levels, colon tissues were homogenated and sonicated in M2 buffer. Three-hundred micrograms of colon protein were used to measure TNF-α, IL-6, and IL-1β levels. BioLegend’s ELISA MAX™ Deluxe Sets for TNF-α, IL-6 and IL-1β were used. The experiments were conducted according to manufacturer’s instructions.

### TUNEL (terminal dexynucleotidyl transferase (TdT)-mediated dUTP nick end labeling) staining

Sections of formalin-fixed, paraffin-embedded tissues were deparaffinized with xylene and rehydrated through graded ethanol. Sections were digested with Proteinase K at 55 °C for 1 h and stained using a TUNEL Apoptosis Detection Kit (FITC) (Yeasen, Shanghai, China) according to manufacturer’s instructions. Ten random fields (200×) were photographed and FITC positive cells were counted. The average number of FITC positive cells per field are presented.

### Measurement of cell death

HT-29 or L929 cells were pretreated with UCF-101 (50 μM) for 1 h, then stimulated with 20 ng/mL TNF-α plus 2 μM Smac mimetic (BV-6) and 25 μM Z-VAD (T/S/Z) for 8 h or 20 ng/mL TNF-α plus 2 μM Smac mimetic (T/S) for 9 h or 1 ng/mL TNF-α plus 25 μM Z-VAD (T/Z) for 3 h, respectively. For PI staining, cells were digested with trypsin containing 0.25 M EDTA, washed with cold 1× assay buffer, stained with PI for 5 min and then analyzed by flow cytometry. For PI/Hoechst staining, cells were stained with PI and Hoechst for 20 min, then photographed with a fluorescence microscope and at least 300 cells were counted. The ratio of PI positive cells (%) = (PI positive cells)/(Hoechst positive cells) × 100%. For cell viability analysis, CCK-8 was add to the well and incubated for 1–2 h and then OD450 was measured using a multi-mode microplate reader (Varioskan Flash, Thermo, USA). Cell viability = (OD_target_ − OD_blank_)/(OD_control_ − OD_blank_) × 100%. Target = cells treated with T/S/Z or T/S or T/Z, control = cells with no treatment, blank = no cells.

### ShRNAs and gene knockdown

HtrA2 shRNA (shHtrA2) and nontarget control shRNA (shNC) constructs were purchased from Cyagen Biosciences (China). Sequences of shRNAs are list in Supplementary Table 2. Lentiviruses were generated by transiently cotransfecting HEK293T cells with the lentiviral expression vector (pLV-shHtrA2) and packaging plasmid (Lenti-X HTX Packaging Mix, Clontech, San Francisco, CA, USA) using Lipofectamine 3000 (Life technologies, USA). Twelve hours after transfection, cells were refreshed with complete growth medium and incubated for another 36 h. The lentiviral supernatants were then harvested and cellular debris was removed by centrifugation at 700 *g* for 10 min. HT-29 cells were then infected with lentiviruses. Knockdown efficiency was determined by immunoblotting. To ensure knockdown efficiency, cells within six generations were used.

### Statistical analysis

Data from at least three independent experiments are shown as the mean ± standard error of the mean (SEM). Unless otherwise noted, the differences between two groups were analyzed by unpaired Student *t* test. Mouse survival curves were constructed using the Kaplan–Meier product limit estimator and log rank (Mantel–Cox) test. Analyses were performed with GraphPad Prism (Version 4.0, USA). *P* < 0.05 was considered statistically significant in all experiments.

## Supplementary information

Supplementary Figures and Figrue legends

Supplementary Figure 1

Supplementary Figure 2

Supplementary Figure 3

Supplementary Figure 4

Supplementary Figure 5

Supplementary Figure 6
