# Supplementary material for: Inhibition of HtrA2 alleviated dextran sulfate sodium (DSS)-induced colitis by preventing necroptosis of intestinal epithelial cells
Source: Cell Death Dis. 2019 Apr 24;10(5):344. doi: 10.1038/s41419-019-1580-7 (PMC6482197; doi:10.1038/s41419-019-1580-7)
Supplement: Supplementary file 1 — Supplementary Figures and Figrue legends [file 41419_2019_1580_MOESM1_ESM.docx]

**Supplementary Table 1. The incidence of bacterial spreading to the spleens of mice.**

| Number of mice | Bacteria in Spleen | | *P* value |
| --- | --- | --- | --- |
|  | Yes | No |  |
| H2O | 0 | 8 |  |
| DSS+DMSO | 3 | 3 | 0.0455 |
| DSS+UCF-101 | 0 | 6 |  |

**Supplementary Table 1. UCF-101 decreased the incidence of bacterial spreading to the spleens of DSS-treated mice.** 3% DSS was administered in drinking water to C57BL/6 mice for 7 days and replaced with fresh water thereafter. UCF-101 (10 μmmol/Kg mice) or DMSO was injected intraperitoneally every day for 8 days. Bacterial load in the spleen was analyzed (see “Material and Methods” for details), the number of mice with or without bacterial spreading to the spleen were shown above.

| **Supplementary Table 2. Sequences of shRNAs** | |
| --- | --- |
| **Name** | **Sequences** |
| shNC | CCT AAGGTTAAGT CGCCCTCGACCGGTCGAGGG CGACTTAACC TTAGG |
| shHtrA2-1 | AGC ACCTGCCGTG GTCTATATCTCGAGATATAG ACCACGGCAG GTGCT |
| shHtrA2-2 | CTG ATCGTCTTCG AGAGTTTCCTCGAGGAAACT CTCGAAGACG ATCAG |

**Supplementary Figures**


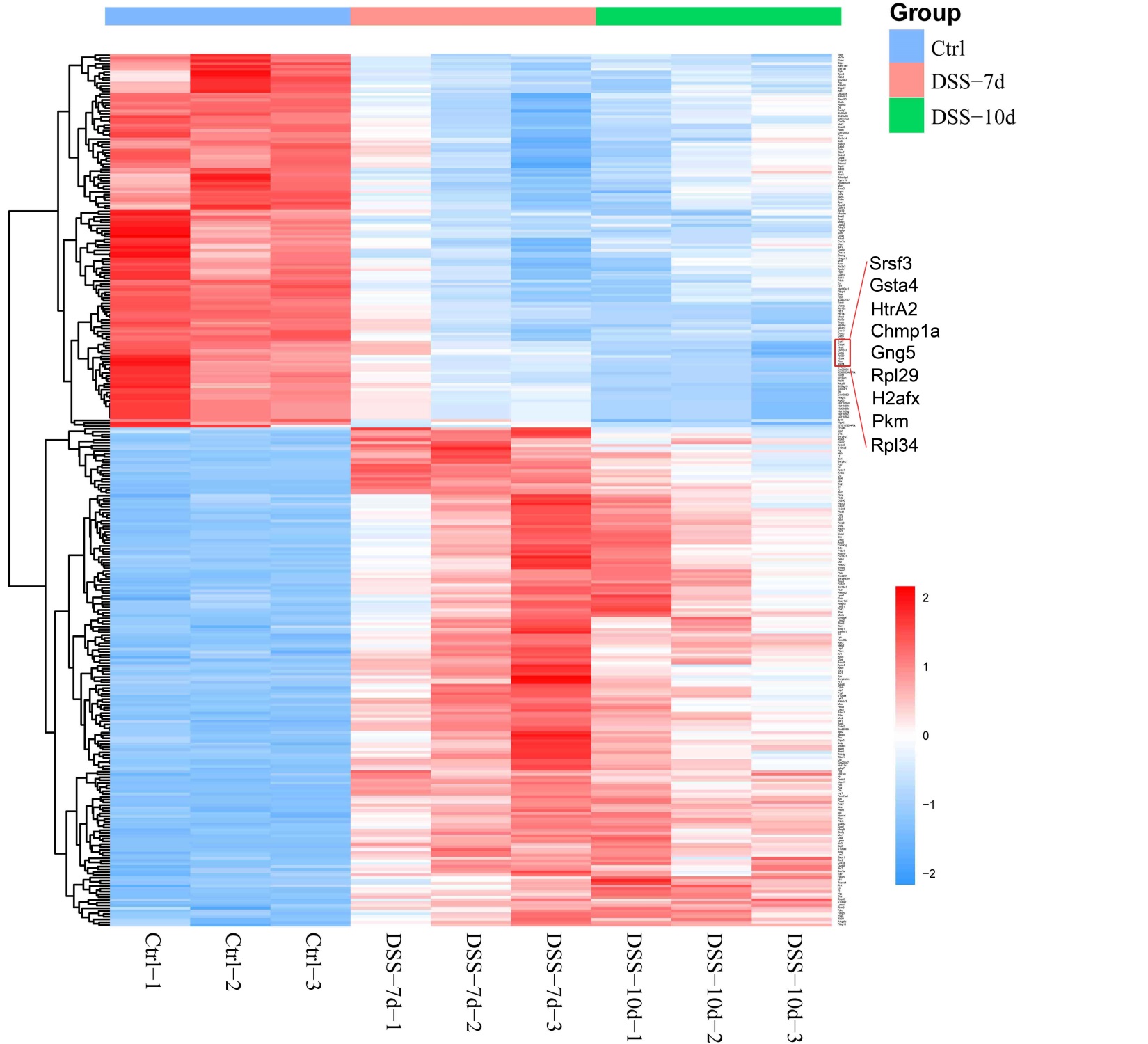


**Figure S1. Differentially expressed proteins in colons of control and DSS treated mice.** 3% DSS was administered in drinking water to C57BL/6 mice for 7 days and replaced with fresh water for the following days. On day 7 and day 10, colons were collected and protein levels were measured by TMT quantitative proteomics. N = 3 mice/group, and 1-3 represent 3 individual mice. Proteins with fold change in a comparison > 1.3 or < 0.77 and unadjusted significance level *P* < 0.05 were considered differentially expressed. Unbiased clustering and cluster-specific enrichment analysis of 315 differentially expressed proteins between each group. The heatmap was drawn using “Heatmap” R package, and the rows were scaled. Z-score is a statistic normalization using ratios of each group through an arithmetic included in “Heatmap” R package. The left branch is called cluster. After the completion of z-score processing, the genes are clustered according to the similarity of expression quantity, and the genes with similar expression rules will be ranked together, while the genes with greater difference in expression patterns will be far away. The red and blue bar on the right is color key, and the value is the normalized Z value (generally -2~2,), which is used to represent the level of expression after normalization. Blue represents the low expression level, while red represents the high expression level.

**
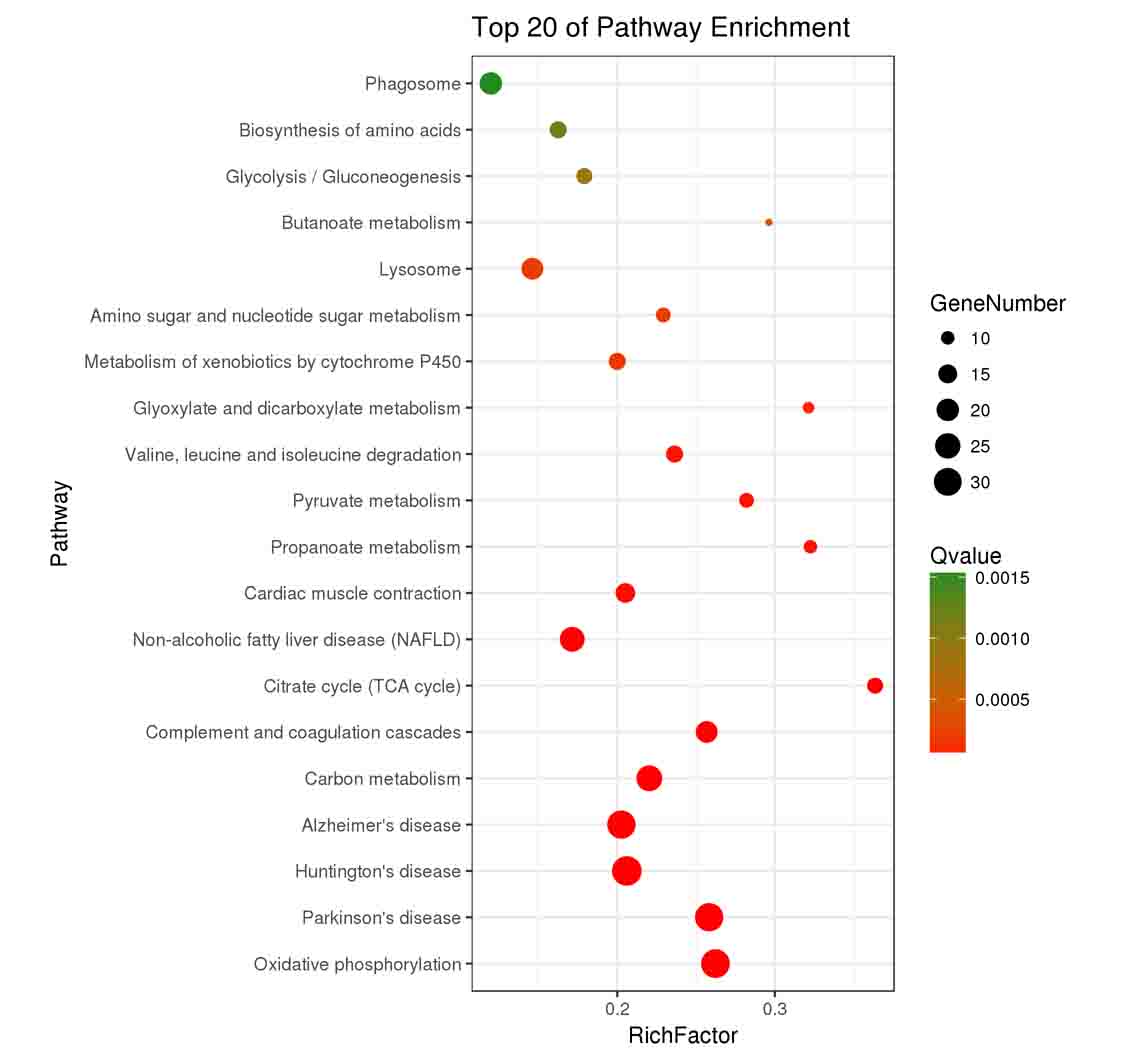
**

**Figure S2. KEGG analysis of differentially expressed proteins in colons of control and DSS treated mice.** 3% DSS was administered in drinking water to C57BL/6 mice for 7 days and replaced with fresh water for the following days. On day 7 and day 10, colons were collected and protein levels were measured by TMT quantitative proteomics. Proteins with fold change in a comparison > 1.3 or < 0.77 and unadjusted significance level P < 0.05 were considered differentially expressed. KEGG analysis of differentially expressed proteins were performed. The enrichment bubble chart shows the first 20 pathways that showed the most significant (most statistically significant) enrichment. Q-value (also called FDR value) is the P-value corrected after multiple hypothesis testing. The value range is 0 to 1, and the closer to 0, the more significant the enrichment. The color of bubbles indicates significance. The redder the color is, the smaller and more significant the q-value is. The size of the bubble represents the number of genes/proteins, the larger the bubble is, the more the number of proteins there are. Rich Factor = n/N, n refers to the number of differentially expressed proteins in the pathway entry, and N represents the total number of proteins located in the pathway entry of all proteins. The larger the Rich Factor is, the higher the enrichment degree will be.


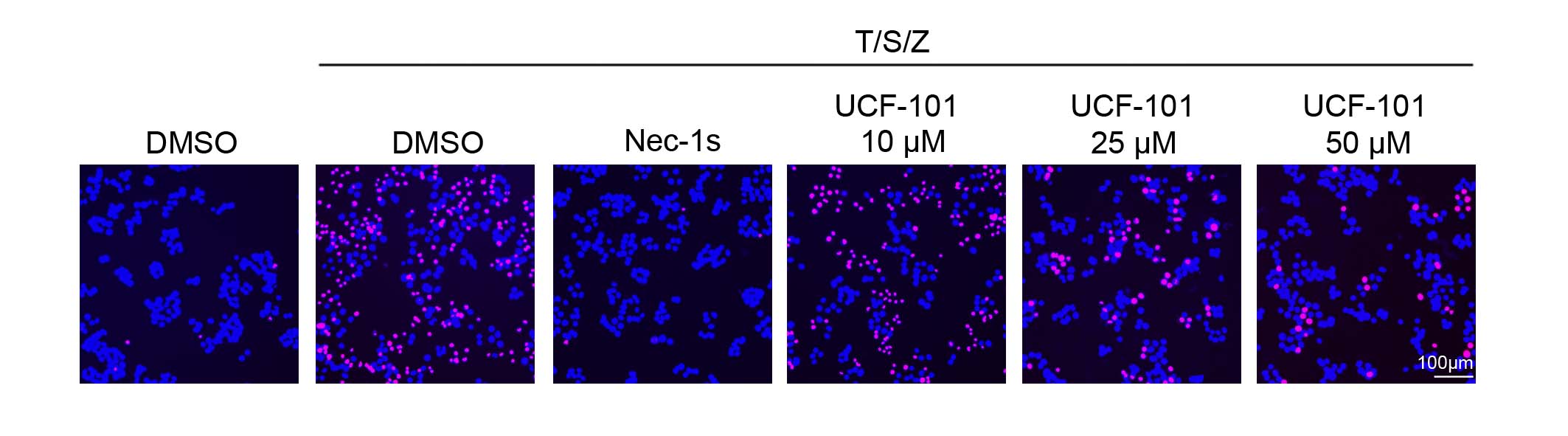


**Figure S3. Representative images of PI/Hoechst staining corresponding to Figure 6.** PI, red; Hoechst, blue. Scale bar, 100 μm.


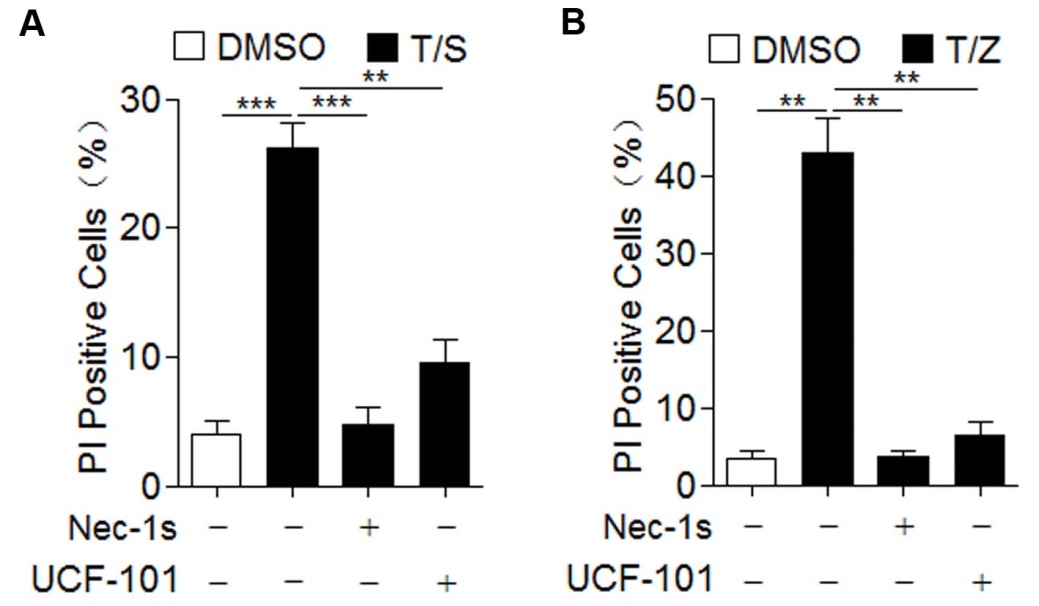


**Figure S4. UCF-101 decreased necroptosis in TNF-α/Smac (T/S) or TNF-α/Z-VAD (T/Z) treated L929 cells.** (A) L929 cells were pretreated with Nec-1s (10 μM) or UCF-101 (50 μM) for 1 hour, followed by stimulation with TNF-α (1 ng/mL)/Smac (2 μM) for 9 hours. PI positive cells were analyzed by PI staining and measured by flow cytometry. (B) L929 cells were pretreated with UCF-101 (50 μM) for 1h, then stimulated with TNF-α (1 ng/mL)/Z-VAD (25 μM) for 3 hours. PI positive cells were analyzed by PI/Hoechst staining. Data are presented as means ± SEM. In (A and B), data are presented as means ± SEM. **, P < 0.01; ***, P < 0.001 (two-tailed unpaired Student’s t test).


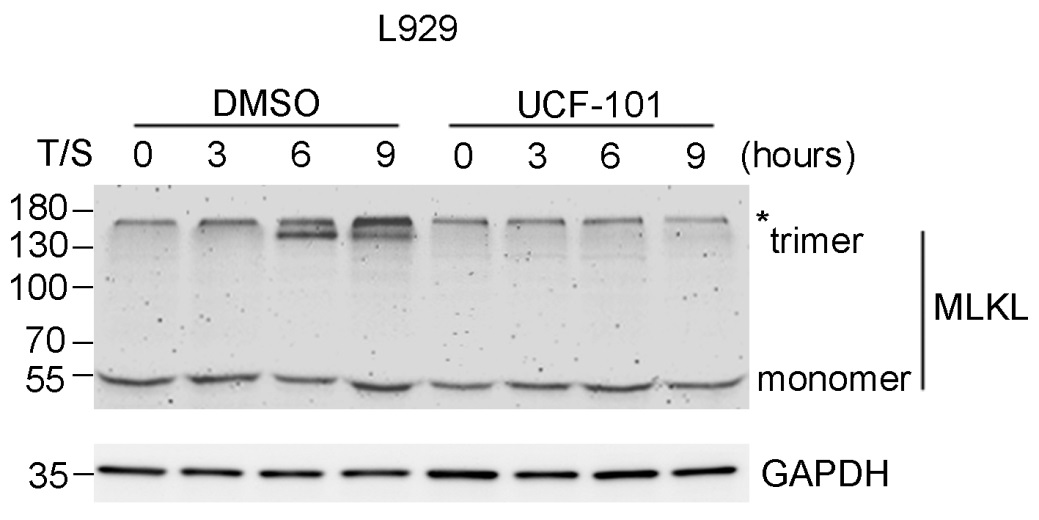


**Figure S5. UCF-101 inhibited formation of MLKL trimer in T/S treated L929 cells.** L929 cells were pretreated with UCF-101 (50 μM) for 1h, then stimulated with T/S for different times as indicated. The cell lysates were resolved on non-reducing gel and MLKL monomer and trimer were detected by immunoblotting with anti-MLKL antibody. GAPDH was used as an internal control. * indicates non-specific band.


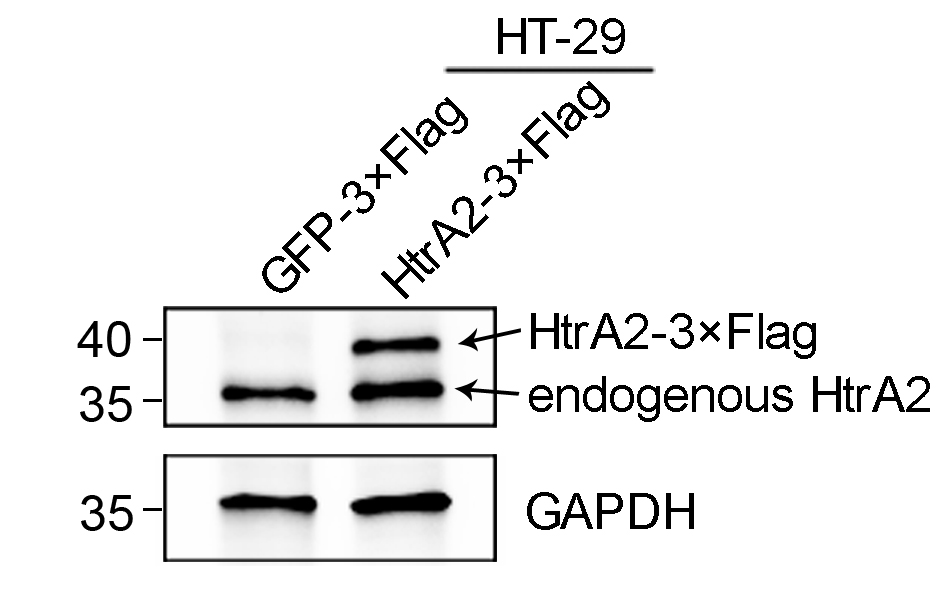


**Figure S6. Stable expression of HtrA2-3×Flag in HT-29 cells (HT-29- HtrA2-3×Flag) by lentivirus.** Expression of endogenous HtrA2 and HtrA2-3×Flag were detected by immunoblotting using HtrA2 antibody, GAPDH as an internal control.
