# Supplementary figures and images for: Inhibition of HtrA2 alleviated dextran sulfate sodium (DSS)-induced colitis by preventing necroptosis of intestinal epithelial cells
Source: Cell Death Dis. 2019 Apr 24;10(5):344. doi: 10.1038/s41419-019-1580-7 (PMC6482197; doi:10.1038/s41419-019-1580-7)

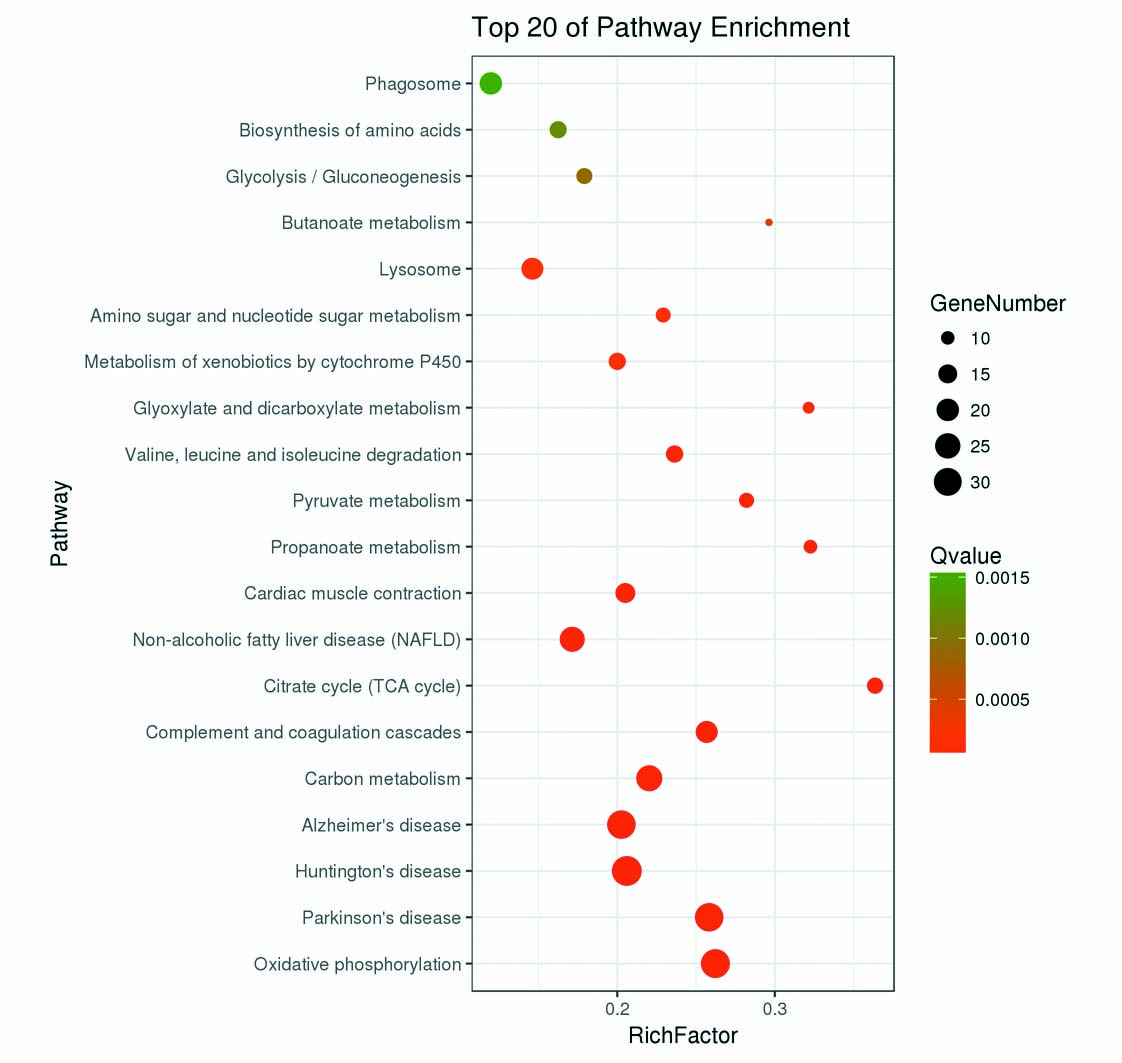

Supplement: Supplementary file 3 — Supplementary Figure 2 [file 41419_2019_1580_MOESM3_ESM.jpg]

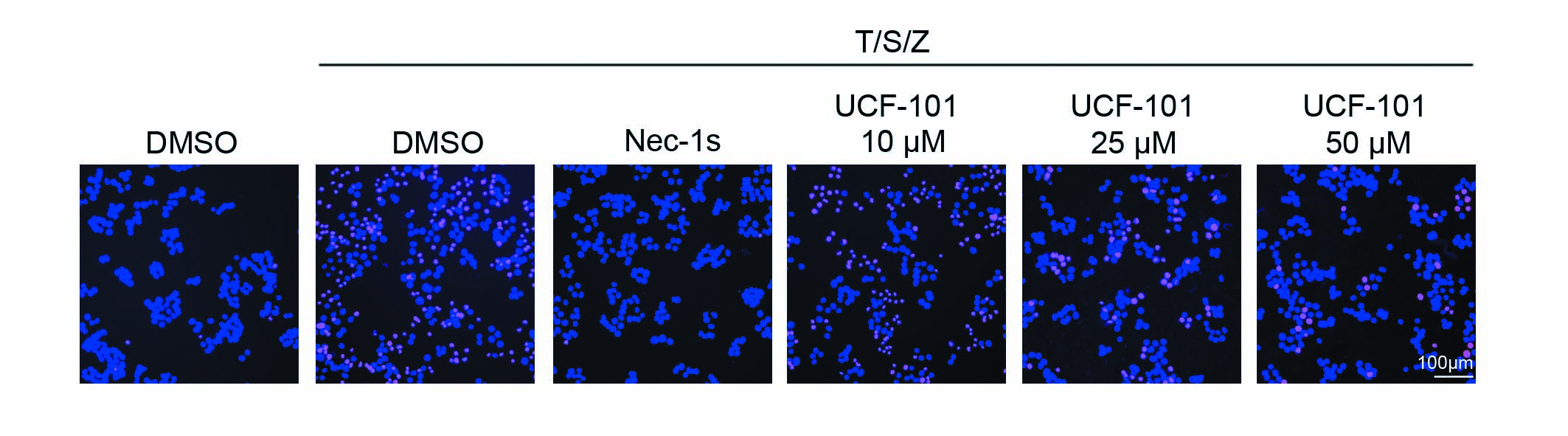

Supplement: Supplementary file 4 — Supplementary Figure 3 [file 41419_2019_1580_MOESM4_ESM.jpg]

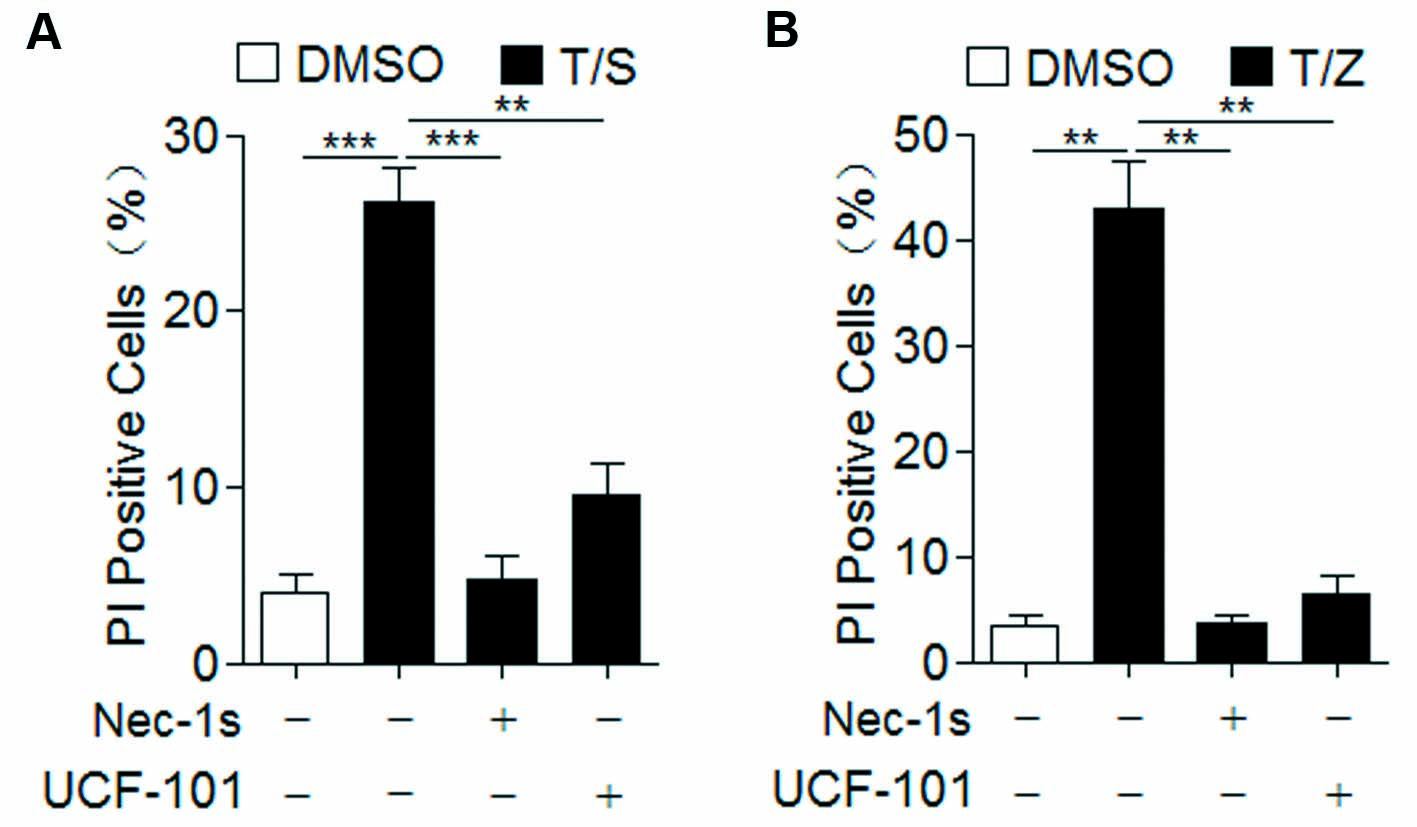

Supplement: Supplementary file 5 — Supplementary Figure 4 [file 41419_2019_1580_MOESM5_ESM.jpg]

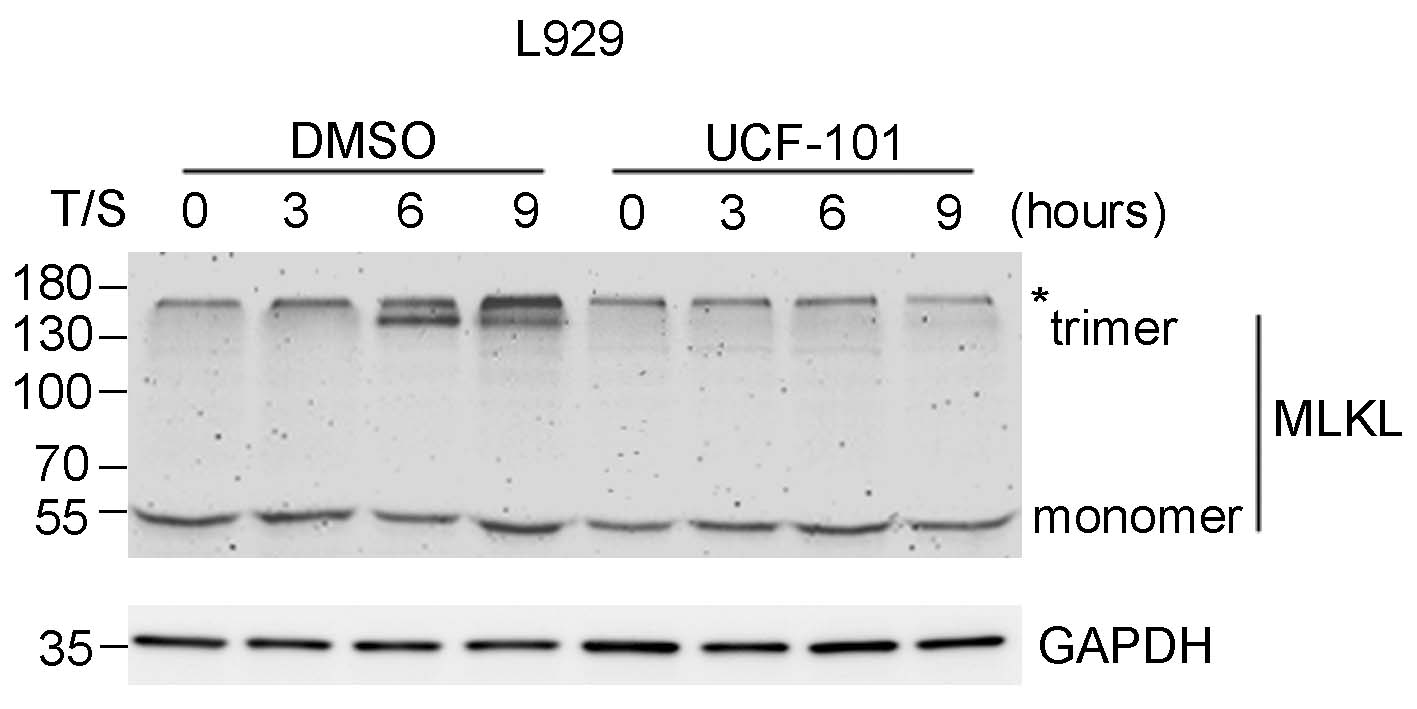

Supplement: Supplementary file 6 — Supplementary Figure 5 [file 41419_2019_1580_MOESM6_ESM.jpg]

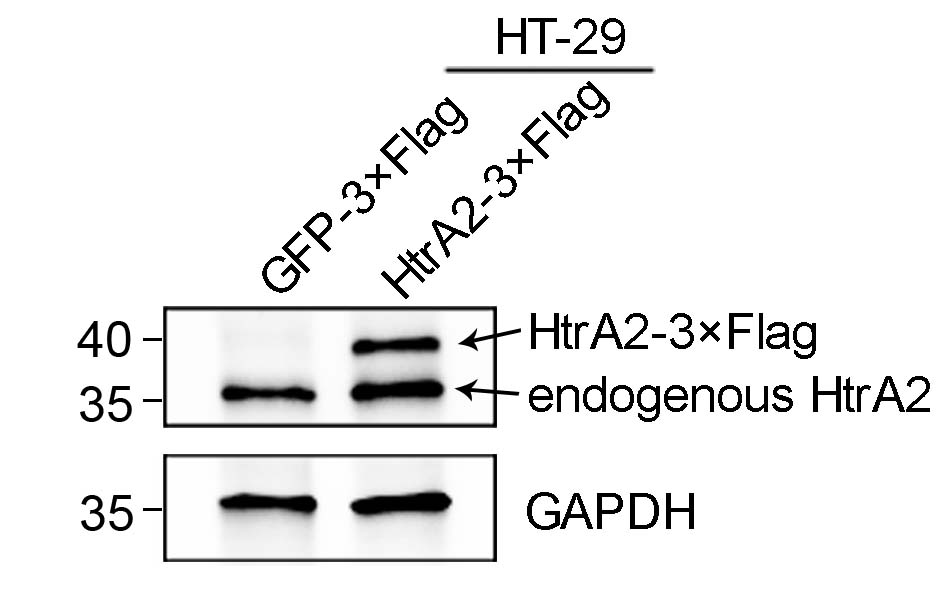

Supplement: Supplementary file 7 — Supplementary Figure 6 [file 41419_2019_1580_MOESM7_ESM.jpg]
